# Challenges and prospects of integrating mental health education in Chinese schools

**DOI:** 10.3389/fpubh.2025.1621170

**Published:** 2025-09-04

**Authors:** Yingmei Shang, Jiao Wang, Haifeng Zhu

**Affiliations:** ^1^Institute of Ideological and Political Education, Northeast Normal University, Changchun, China; ^2^School of International Studies, Northeast Normal University, Changchun, China

**Keywords:** mental health education, student mental development, Chinese schools, challenges, prospects

## Abstract

China has long been committed to promoting students’ mental well-being by integrating mental health education (MHE) across primary, secondary, and higher education. This paper offers a comprehensive analysis of how MHE is integrated in Chinese schools. It first examines the current state of integration through a review of policy and relevant literature. It then explores the key challenges across four dimensions: institutional mechanisms, educational practices, targeted interventions, and teacher workforce development. Finally, the paper discusses future directions for advancing MHE in Chinese schools, including updating educational philosophies, strengthening institutional frameworks, building holistic student support systems, and enhancing teacher training, ultimately aiming to promote the high-quality development of MHE in China.

## 1 Introduction

In recent years, poor mental health has emerged as a significant global social challenge, drawing increasing international attention. In China, the prevalence of mental health issues has gradually expanded beyond professional populations to include students across all educational stages from primary schools to universities. A comparative analysis by Yu and Huang (1) revealed notable differences in depression detection rates across these stages: 13.5% among primary school students, 23.9% among junior secondary students, 28.0% among senior secondary students, and 20.8% among university students. These data suggest a correlation between academic progression and heightened psychological strain, especially during the senior secondary stage, where students encounter the intense pressures of the high-stakes college entrance examination. In particular, detection rates of poor mental health among this group are highest for depression (28.0%), followed by anxiety (26.3%), self-injury (23.0%), sleep problems (22.8%), suicidal ideation (17.1%), somatization (9.8%), suicide planning (6.9%), and attempted suicide (2.9%) (2). Contributing factors may include deficits in self-regulation and the lack of timely educational support or psychological interventions, which can exacerbate the vulnerability of at-risk students.

A survey report by the United Nations Children’s Fund (UNICEF) revealed that mental health conditions “account for 16% of the global burden of disease and injury among the world’s 1.2 billion adolescents aged 10–19” and estimated that “10–20% of adolescents experience mental disorders” (3). In China, a national survey reported in 2021 found that the prevalence of mental disorders among primary and secondary school students aged 6–16 had reached 17.5% (4). Furthermore, the Report on National Mental Health Development in China (2021–2022), released by the Chinese Academy of Sciences in 2022, surveyed nearly 80,000 college students and found that approximately 21.48% suffer from depression, while 45.28% experience anxiety (5).

In light of these escalating concerns, the implementation of MHE in schools has become a key component of China’s strategic efforts to promote students’ mental well-being. MHE in schools refers to the provision of comprehensive psychological education within educational settings. It encompasses formal mental health curricula, school-wide cultural, artistic, and athletic activities aimed at fostering psychological resilience, counseling services, as well as targeted interventions for students experiencing psychological distress. The primary objectives of MHE are to enhance students’ overall mental well-being, prevent the onset of psychological problems and crises, and provide timely support and interventions for those who are experiencing or at risk of developing mental disorders.

To more effectively address the mental health challenges faced by students, the Chinese government has progressively shifted from fragmented, isolated efforts to a more systematic and integrated model of MHE spanning all educational stages, from primary to higher education. A key milestone in this transformation is the Special Action Plan for Comprehensively Strengthening and Improving Student Mental Health in the New Era (2023–2025) (hereafter Special Action Plan) issued in 2023. This policy institutionalizes a fully integrated MHE system across primary, secondary, and higher education in China (6). Chinese scholars have also highlighted the importance of such an integrated approach. For example, Yu and Chen (7) advocated for MHE as a comprehensive framework for analyzing and addressing students’ mental health needs, emphasizing both “the application of MHE across different educational stages, from nurseries to higher education” and “the reasonable integration of mental health courses with other related disciplines.”

This study aims to comprehensively examine the integration of MHE in Chinese schools through a multi-faceted investigation. First, it presents the current state of MHE integration in Chinese schools by conducting a comparative analysis of relevant policies in China and other countries, alongside a review of the domestic literature. Next, it identifies and analyzes the key challenges facing current integration efforts. Based on these findings, the study explores future directions for advancing integrated MHE in Chinese schools, with the ultimate goal of enhancing students’ mental well-being.

## 2 Comparative analysis of MHE policies in China and other countries

The modern concept of school-based MHE originated in the United States and subsequently developed rapidly in many other developed countries (8). International approaches to integrating MHE across primary, secondary, and higher education stages demonstrate distinct policy emphases. For instance, the United States’ 2022 National Health Education Standards (3rd Edition): Model Guidance for School Curriculum and Instruction provides nationwide guidance for schools, while Japan’s White Paper on Education, Culture, Sports, Science, and Technology (2022) offers a comprehensive compilation of theoretical frameworks, educational philosophies, and practical teaching methods for students from primary to high schools.

China’s MHE initiatives began later than those of many developed countries but have progressed rapidly in recent decades. The Chinese government has issued a series of policy documents and directives aimed at integrating MHE across all stages of education (see Table 1). These efforts reflect a high degree of systematicity and continuity in building a cohesive MHE policy framework spanning primary, secondary, and higher education. For example, since the 1999 issuance of Several Opinions on Strengthening MHE in Primary and Secondary Schools, MHE policies for these stages have been revised and refined in subsequent years, specifically in 2002, 2012, 2018, and 2021. Moreover, in recent years, the government’s focus has expanded from primary and secondary education to a more comprehensive approach that includes all educational stages, thereby establishing an integrated MHE system that forms a continuous policy chain from primary schools to higher education.

**Table 1:** Policy documents and key meetings on MHE integration across educational stages.

Issuance date	Documents or meeting title	Issuing authority
August 13, 1999	Several Opinions on Strengthening MHE in Primary and Secondary Schools	Department of Basic Education, Ministry of Education (Document No. 13 of 1999)
March 16, 2001	Opinions on Strengthening MHE for College Students in General Higher Education Institutions	Department of Social Sciences, Ministry of Education, Social Affairs (Document No. 1 of 2001)
August 1, 2002	Guidelines for MHE in Primary and Secondary Schools	Department of Basic Education, Ministry of Education (Document No. 14 of 2002)
December 11, 2012	Guidelines for MHE in Primary and Secondary Schools (Revised in 2012)	First Division of Basic Education (Document No. 15 of 2012)
July 16, 2018	Guidelines for MHE in Higher Education Institutions	CPC Leadership Group of the Ministry of Education (Document No. 41 of 2018)
July 12, 2021	Notice on Strengthening Student Mental Health Management Work from the Ministry of Education	Official Letter from the Ideological and Political Education Office (Document No. 10 of 2021)
April 27, 2023	Notice on the Issuance of “the Special Plan for Comprehensively Strengthening and Improving Student Mental Health Work in the New Era (2023–2025)” by the Ministry of Education and Other Sixteen Departments	Department of Physical, Health and Arts Education, Ministry of Education (Document No. 1 of 2023)
February 21, 2024	The First Plenary Session of the National Student Mental Health Work Consultative Committee	Ministry of Education

China has developed a “pyramid-style” policy system for MHE, characterized by top-level strategic planning that offers comprehensive guidance and robust institutional support. A significant milestone in this policy evolution is the 2023 Special Action Plan, which emphasizes multi-departmental collaboration, integration of medical and educational resources, and coordinated efforts among families, schools, and communities. This plan signifies that student mental health has been elevated to a national strategic priority, occupying a more prominent and critical position in policy-making.

An analysis of policy documents and key national conferences reveals that China places significant emphasis on MHE for students across all educational stages, integrating strategic planning, top-level design, and practical implementation. Supported by a strong policy framework, MHE has generally been implemented with measurable progress in primary, secondary, and higher education. However, several challenges persist, including uneven policy enforcement, fragmented implementation in which institutions often operate in isolation, and the lack of a well-coordinated, government-led, multi-sectoral collaboration model necessary for delivering holistic and integrated MHE.

## 3 Overview of Chinese domestic research on the integration of MHE in student development

**Figure 1:**
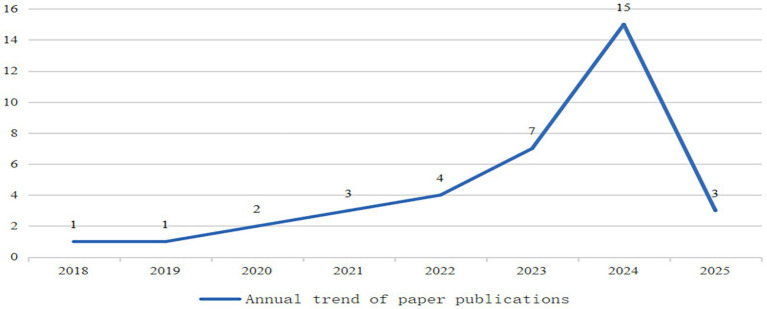
Annual trends in publications containing the keyword “integrated MHE in primary, secondary, and higher education.”

As of April 5, 2025, using the China National Knowledge Infrastructure (CNKI) as the research database and the keyword “integrated MHE in primary, secondary, and higher education,” we identified 36 relevant papers. As shown in Figure 1, scholarly interest in the integration of MHE within the Chinese educational system began to emerge around 2018, followed by a steady increase in research output, reaching 15 publications by 2024. Researchers have examined this integration from diverse perspectives, including disciplinary and theoretical frameworks, different student age groups, empirical studies, and service domains. They have covered a broad range of topics, including current situation analysis, management systems, development of school psychologists, curriculum design, textbook content, organized activities, archive construction, monitoring and intervention strategies, and policy recommendations.

Among these studies, Yu’s (7) research team is widely recognized for producing some of the most comprehensive, detailed, and authoritative works in the field of integrated MHE. Between 2020 and 2021, the team published four influential papers examining the necessity of integrating MHE in schools from multiple perspectives, including curriculum design, moral education, personality development, and practical implementation. In particular, Yu and Chen argued that curriculum design should incorporate ideological guidance, curriculum development, and management strategies to optimize MHE effectiveness. Yu (9) further highlighted the importance of addressing broader issues such as social phenomena, family-school collaboration, and the monitoring of moral cognition to support students’ moral development. Additionally, Yu and Zhang (10) examined the psychological and social challenges faced by students and advocated for “an overall plan and framework for the goals and details of MHE across all educational stages to support their personality development.”

However, since students face distinct mental health challenges at different educational stages, the broad assumptions underlying this overall plan and framework may pose challenges for practical implementation. To address this, Yu (11) recommended that “MHE in different stages should be tailored to courses, activities, counseling, and screening, adopting an effective integrated educational approach that aligns with students’ developmental needs.”

The significance of these studies lies in their potential to enhance students’ mental health awareness, inform government policies, and guide the development of effective interventions that promote both well-being and academic success. Moreover, they offer valuable insights for global mental health research. However, while they have primarily focused on theoretical frameworks, social factors, and individual mental health issues, comparatively little attention has been paid to the role of administrative bodies—such as governments and schools—in addressing these challenges.

## 4 Challenges in integrating MHE in Chinese schools

It is widely recognized that adolescents’ mental health forms the foundation for their personal development, academic achievement, and long-term career prospects. The Chinese government has placed significant emphasis on student mental health, developing increasingly comprehensive policies and guidelines. Student mental health education (SMHE) has thus achieved an unprecedented status as a national strategic priority. Notably, the 2023 Special Action Plan articulates guiding ideologies, fundamental principles, and clear objectives, underscoring the strategic and systematic nature of policy formulation. Furthermore, its key initiatives and safeguard measures highlight the plan’s comprehensiveness, professionalism, and strong commitment to effective implementation.

However, the integration of MHE in Chinese schools, as outlined in the 2023 Special Action Plan, faces significant challenges due to the complexity of students’ mental health issues and various social factors. Identifying these challenges is both urgent and essential to facilitate effective implementation. This study focuses on key obstacles related to institutional mechanisms and coordination, systematic planning and execution, the identification and classification of mental health issues, and the training and shortage of school psychologists. A comprehensive analysis of these four main challenges is crucial for establishing a solid foundation to accurately evaluate the outcomes of MHE integration and to ensure targeted, effective policy implementation.

### 4.1 Ineffective coordination in institutional mechanisms across educational stages

First, the institutional management mechanism across different educational stages lacks effective coordination, hindering the comprehensive implementation of SMHE. Although the 2023 Special Action Plan calls for collaboration among various institutions to deliver systematic MHE, in practice, many institutions fail to fully adhere to these guidelines, resulting in inadequate communication and coordination. A major challenge is the insufficient collaboration among schools, education bureaus, medical institutions, and community organizations, which disrupts the integration of MHE across all educational stages. Addressing this issue requires a systematic governance approach that prioritizes the establishment of strong multi-departmental coordination mechanisms and reinforces cooperation among schools, families, and society to ensure the effective delivery of SMHE.

Furthermore, the integration of MHE across primary, secondary, and higher education has not been sufficiently addressed in existing policy documents. Key components—including guiding ideologies, fundamental principles, objectives, and major tasks—remain inadequately defined. In addition, the lack of unified, professional implementation standards, coupled with insufficiently informed and effective policy support, poses significant challenges. Consequently, educational institutions may encounter developmental bottlenecks that hinder the advancement of MHE.

A common challenge lies in the incomplete or ineffective transfer of student psychological portfolios across educational stages, resulting in discontinuities in tracking the developmental trajectory of students’ mental health by school mental health professionals. Liu (12) noted that student mental health portfolios in primary and secondary schools often lack essential data, which are only supplemented when students enter higher education. As a critical component of MHE, psychological interventions play a vital role in preventing the exacerbation of mental health problems among students who have already shown signs of distress. If student psychological portfolios were able to comprehensively document mental health progression beginning in primary school, educators at subsequent stages would be better equipped to provide timely and targeted interventions. However, safeguarding the confidentiality of sensitive information during the transfer of these portfolios poses a significant challenge. To address this, educators at all stages must integrate psychological portfolios into a secure and confidential student file system and strengthen procedural oversight of portfolio transfers, thereby protecting students from potential prejudice or discrimination based on their mental health history.

### 4.2 Gaps in systematic planning and implementation across educational stages

In addition to management-related challenges, the lack of continuity, coherence, and systematic planning in MHE across different educational stages significantly limits its effectiveness, highlighting the need for clearer guidance and stronger policy enforcement. In response to evolving societal needs, the Chinese government has issued multiple policies since 1999 (see Figure 1), each outlining specific plans for school-based MHE. Among these, the 2023 Special Action Plan stands out as the most comprehensive and systematic, offering explicit guidance on curriculum requirements, implementation strategies, and student-centered interventions.

Although the Chinese government has issued multiple policy documents emphasizing SMHE, two key challenges persist in systematic planning and concrete implementation. On the one hand, from an overarching educational perspective, while continuity, progression, and differentiation of MHE across educational stages are acknowledged, there remains a lack of clear and actionable guidance. As a result, MHE at various stages suffers from deficiencies in continuity and coherence, particularly in areas such as curriculum development, textbook compilation, activity design, and the training of school psychologists. In some instances, gaps even exist between educational stages. This is reflected in the repetition of content across stages and the tendency to embed MHE within broader subjects such as moral and political education. Consequently, MHE often receives insufficient attention or is neglected altogether.

On the other hand, from the perspective of individual students, the fragmentation of educational resources hinders the formation of a cohesive support system. Consequently, psychological confusion, mental health issues, and mental disorders that arise during students’ academic progression often remain unaddressed in a timely manner, leading to their accumulation over time. This fragmentation diminishes the overall relevance and effectiveness of MHE across different educational stages. Therefore, it is essential to introduce a series of complementary policies grounded in a top-level institutional framework to ensure more effective and coordinated implementation.

### 4.3 Inconsistent and incomplete implementation of targeted interventions across educational stages

The precise identification and classification of students with mental disorders at various educational stages are critical. Students face distinct psychological challenges throughout their academic journey. According to the Report on National Mental Health Development in China (2021–2022), published by the Chinese Academy of Sciences, approximately 21.48% of students suffer from depression, while 45.28% experience anxiety. These figures highlight the urgent need for accurate identification and classification of mental health issues. However, inconsistencies and gaps in assessment persist across different educational stages, resulting in overlooked areas of mental health support. Consequently, many students may encounter preventable psychological crises due to a lack of timely, effective, and scientifically informed interventions. Failure to adequately address these issues can have profound negative repercussions for families, schools, and society at large.

Identifying students with mental disorders and providing targeted interventions is crucial. However, many schools across educational stages lack the capacity to effectively recognize and address the diverse mental health needs of their students. Schools must adopt proactive measures to prevent the onset of mental disorders, offer timely treatment for at-risk students, and deliver appropriate care for those already diagnosed with mental health conditions. Furthermore, the Symptom Checklist-90 (SCL-90), a widely used psychological assessment tool, was originally designed for individuals with psychiatric disorders. When applied to the general student population, it may overemphasize pathological symptoms while overlooking the normal psychological states of students, thereby compromising the comprehensiveness and accuracy of mental health evaluations (13). Overreliance on mental health screenings or singular test results weakens the thoroughness and reliability of student mental health portfolios.

Students with persistent mental disorders often continue attending school despite their condition, which underscores the urgent need for schools to strengthen identification and support systems for these individuals. Without timely intervention, their mental health conditions may deteriorate as they advance through different educational stages, potentially leading to severe negative consequences. The circumstances surrounding these students are often complex, involving both general and individualized factors. While the manifestations and underlying causes vary across educational stages, they are typically linked to a combination of influences such as family dynamics, parent–child relationships, interpersonal interactions, emotional stress, and psychological pressure.

One significant challenge in MHE is that students diagnosed with a mental disorder may experience feelings of shame, which can further exacerbate their mental health conditions. Moreover, students, parents, and even teachers often lack a clear understanding of mental distress and mental disorders, frequently holding outdated beliefs and struggling to address these issues openly. Moreover, once an individual is assessed or diagnosed with mild, moderate, or severe mental disorders through psychological evaluations or clinical institutions, they may suffer from the so-called “labeling effect,” leading to negative self-perceptions and social stigma. The stigmatization of mental disorders has persisted across cultures and throughout history (14). Note that psychological assessments and diagnoses serve as objective indicators and reference points rather than absolute determinations. Mental health conditions are inherently dynamic and subject to change; with professional treatment, evidence-based interventions, and personal resilience, they can often be effectively managed or even fully overcome.

### 4.4 Insufficient allocation and poor coordination of MHE personnel across educational stages

Many Chinese schools across educational stages face significant challenges in the allocation, quality, and coordination of MHE personnel. The shortage of qualified professionals not only diminishes the overall effectiveness of MHE but also places excessive workloads and pressure on existing staff. As a result, their own mental well-being, motivation, and professional development may suffer, undermining both individual career progression and the systematic, coherent advancement of MHE programs.

There is a notable shortage of MHE personnel in schools. The Guidelines for MHE in Primary and Secondary Schools (2022) mandate that each school employ at least one full-time mental health professional, with the recommended ratio of professionals to students gradually increasing over time. In some regions, this ratio is set at 1:1,000, while for boarding schools, it is 1:750. Meanwhile, the Guidelines for MHE in Higher Education Institutions (2018) specify that universities should maintain a professional-to-student ratio of no less than 1:4,000 and employ at least two full-time MHE professionals. The primary responsibilities of MHE personnel include promoting psychological knowledge, identifying potential issues, providing MHE, and implementing preventive interventions. Therefore, each educational stage should be staffed with dedicated professionals in accordance with these guidelines to effectively advance MHE in schools.

In practice, only a limited number of schools meet the requirements for employing full-time MHE professionals. Survey data indicate that 29.09% of schools in the Guangxi Zhuang Autonomous Region lack full-time mental health professionals, 37.27% have one, 11.82% have two, 8.18% have three, and 13.64% employ more than three full-time professionals (15). These data highlight the uneven distribution of MHE personnel across schools in Guangxi. Importantly, this issue is not unique to Guangxi but is a widespread challenge across multiple provinces in China. To address this imbalance, the Guangzhou Municipal Education Bureau provides valuable national insights through its pioneering local legislation. The Regulations on the Promotion of Mental Health for Primary and Secondary School Students in Guangzhou, the first local legislation in China specifically aimed at promoting students’ mental health, officially came into effect in November 2024. Under this framework, all primary and secondary schools in Guangzhou maintain 100% staffing of full-time psychological counselors, all of whom have received professional training in MHE (16).

The faculty team responsible for SMHE primarily comprises full-time professionals, supplemented by part-time instructors. However, there is a significant shortage of full-time professionals in terms of both quantity and experience, making it difficult to meet the demands of effective MHE. Additionally, part-time instructors, who often juggle responsibilities such as routine student affairs and general course instruction, typically lack the time and capacity to improve their psychological expertise or actively participate in MHE efforts. Their limited understanding of psychology hampers their ability to deliver MHE effectively. The absence of systematic psychological training and technical skills further restricts their capacity to accurately identify, assess, and intervene in students’ mental health issues. Although professional training is essential for school psychologists and educators, most schools lack adequate training opportunities, further exacerbating the challenge of ensuring high-quality MHE.

There is insufficient continuity in the training and deployment of MHE professionals across educational stages. Due to limitations in time and resources, mental health professionals at various stages of the education system have few opportunities for communication, interaction, and collaboration. Consequently, MHE curricula and activities often operate in isolation at each stage, lacking a seamless progression that is crucial for building a cohesive and continuous MHE framework. This fragmentation leads to two major issues: first, limited information exchange can result in redundancy in teaching content or gaps in critical knowledge, diminishing the specificity and effectiveness of instruction; second, the scarcity of integrated professional development opportunities undermines the coherence and systematic growth of school psychologists’ skills and competencies.

In conclusion, although China has made substantial progress in developing MHE policies, significant challenges persist in their practical implementation. Key obstacles include insufficient coordination across educational stages, inadequate professional support, and gaps in addressing the diverse and evolving psychological needs of students. To overcome these issues, it is essential to establish a comprehensive, top-down institutional framework that promotes multi-level collaboration and resource integration. Coupled with targeted, developmentally appropriate interventions, such a framework will be critical to achieving the effective and seamless integration of MHE throughout all stages of education.

## 5 Prospects of integrating MHE in Chinese schools

SMHE has become a global priority, with many countries integrating it into their national strategies. Governments have issued specific policy documents that reflect high-level national planning in areas such as curriculum development, textbook design, and implementation across various educational stages. For instance, the United States has established standardized school-based MHE through formal guidelines, specialized textbooks, and targeted courses. Similarly, Japan has developed tailored educational materials and textbooks that correspond to the developmental trajectories and educational needs of students at different stages.

In 2023, MHE in China was officially elevated to the status of a national strategy, accompanied by a series of specialized policy documents designed to guide its implementation. Specific provisions have been established to ensure a coordinated framework for MHE across all educational stages. However, amid ongoing societal changes and emerging demands, several challenges persist beyond the previously identified issues of continuity in implementation. These challenges include insufficient precision and comprehensiveness in policy execution, inadequate coordination among government agencies, schools, families, and society, as well as shortcomings in the training and professional development of mental health practitioners. Additionally, there remains a lack of awareness and competency in fostering a holistic approach to students’ mental well-being, limited scope and depth in academic research, and insufficient innovation within MHE. To effectively address these challenges in integrating MHE across primary, secondary, and higher education, targeted efforts must focus on four key areas: updating conceptual frameworks, optimizing institutional mechanisms, constructing a comprehensive educational system, and strengthening the development and support of mental health professionals.

### 5.1 Updating conceptual frameworks

The integration and continuity of MHE across primary, secondary, and higher education represent not only a fundamental pillar of educational development but also a complex, strategic initiative requiring sustained and coordinated efforts. The growing emphasis on SMHE underscores the necessity of adopting a holistic, student-centered approach that nurtures students’ mental well-being throughout every stage of development. Realizing this vision demands a paradigm shift in educational philosophy that embraces innovative conceptual frameworks with both strategic foresight and systemic thinking. Effective MHE must be designed and implemented with a long-term perspective, harmonizing with broader educational goals while equipping students with the psychological resilience and emotional competencies essential for personal growth and academic achievement. This approach should thoughtfully balance preventive interventions with proactive guidance, ensuring that these dimensions reinforce each other and are seamlessly integrated within the overall MHE framework.

In practical terms, this entails two key perspectives. First, a problem-oriented approach should be adopted to address the needs of students experiencing mental health difficulties through targeted interventions. This requires a scientifically informed and professionally guided understanding of critical aspects such as early prevention, intervention for emerging issues, treatment of existing conditions, ongoing support for students with persistent psychological challenges, and the reduction of stigma associated with mental health conditions. A proactive and inclusive stance is essential, ensuring tailored interventions that accommodate diverse psychological needs. The stigmatization of mental health issues among students can be effectively reduced through transdiagnostic interventions, which “have the potential to provide significant benefits by targeting a wider range of mental aspects and addressing co-occurring disorders in a more comprehensive manner” (17). By identifying and addressing the shared core symptoms underlying various mental disorders, transdiagnostic interventions enable broad-spectrum strategies that promote mental health literacy, emotional and behavioral regulation, and value development. This comprehensive framework is better positioned to meet the diverse needs of students. Empirical evidence suggests that transdiagnostic interventions yield a small to medium overall effect size and are more effective than disorder-specific approaches in reducing stigma associated with mental health issues (18). Second, a developmental perspective should be adopted to effectively support students’ mental health. This involves integrating MHE with students’ career development, vocational interests, and life experiences. Developmental MHE should be informed by principles from positive psychology and political education, fostering a proactive approach that supports students’ overall well-being, happiness, and personal growth. Given that senior secondary school students exhibit the highest rates of depression, it is recommended that MHE at this stage be closely integrated with career education through a structured 12-week series of group counseling sessions. Each session can focus on a specific thematic module, such as Exploring Career Interests, Simulating Stressful Situations, or Developing Strength-Based Competencies. Employing interactive techniques such as role-playing and sandplay simulations, the program can foster students’ career identity while simultaneously strengthening their stress management and coping skills.

### 5.2 Optimizing institutional mechanisms

To effectively integrate MHE across primary, secondary, and higher education, it is essential to focus on two key dimensions. The first dimension emphasizes students’ physical and psychological developmental characteristics at each educational stage, as well as adherence to the principles of educational progression. This requires ensuring seamless integration across critical areas such as curriculum design, textbook content, practical activities, and student mental health portfolios throughout the different stages.

Taking student mental health portfolios as an example, their establishment is a highly specialized process that must align with both the developmental characteristics of students at different educational stages and established professional standards and procedural requirements. Mental health issues vary in nature and needs across these stages. The primary and secondary school years represent a critical period for individual development and the formation of mental health. During this stage, students face multiple pressures related to physical and psychological maturation, academic demands, and social challenges. A comprehensive and systematic collection and documentation of students’ mental health information at this stage enables educators to gain an in-depth understanding of their personality traits, life experiences, and psychological developmental trajectories. This facilitates timely identification and intervention during the optimal window for addressing mental health concerns. Moreover, the professionalized development and integration of mental health portfolio systems across educational stages establish a continuous tracking and support mechanism for individual students. This significantly enhances the efficiency of early detection, prevention, and intervention for students experiencing mental health difficulties, thereby creating a rigorous and well-structured feedback loop in MHE.

The second dimension necessitates adherence to the professional requirements and operational characteristics of different sectors. This involves fostering effective coordination and integration among various agencies, including government bodies, schools, families, and society at large, as well as key institutions involved in youth development, such as education authorities, women’s organizations, trade unions, civil affairs departments, sports organizations, community services, mental health institutions, and media and publicity departments. Strengthening collaboration across these diverse entities promotes a synergistic approach to MHE, thereby enhancing the overall effectiveness and reach of MHE initiatives.

### 5.3 Integrating MHE into a holistic student development framework

Given that the scope and depth of MHE extend beyond conventional paradigms, its fundamental objective is the holistic development of students. In the current context of strengthening the education system and advancing high-quality educational development, it is essential to align MHE with the developmental characteristics and educational trajectories of Chinese students. This alignment requires adherence to the principles of systematic and structured implementation, innovation, and localization, aiming to establish a comprehensive MHE system that reflects distinctive Chinese characteristics across all educational stages.

In particular, efforts should be concentrated on two key dimensions. First, it is essential to uphold systemic and structured educational approaches. MHE at different educational stages follows inherent patterns and developmental characteristics that must be recognized as the foundation of educational practice. This requires strict adherence to the principles of educational theory and student development to ensure coherence and professionalism in curriculum design, textbook compilation, and activity planning across all stages. The ultimate goal is to achieve systematic integration and continuity in MHE. To this end, alignment with China’s national strategic priorities is crucial, including strengthening the education system, promoting moral and intellectual growth, enhancing family and societal well-being, and supporting the holistic development of students.

The overarching framework and implementation strategies should emphasize five key aspects: developing comprehensive talent cultivation models that integrate cognitive, emotional, intellectual, and social competencies; designing a structured and progressive mental health curriculum that addresses stress management, person-centered interventions, and environmental supports, while being tailored to the developmental characteristics of each educational stage (19); establishing diverse and interactive activities aimed at alleviating academic pressure and promoting students’ physical and mental health; creating high-quality, research-informed textbooks that reflect students’ developmental needs and cultural contexts; and innovating pedagogical methodologies by incorporating psychological theories, utilizing technological tools, and enhancing teacher training. These elements must be carefully crafted to ensure smooth transitions and professional coherence across educational stages, thereby fostering a systematic MHE approach that is both scientifically rigorous and practically effective.

Second, it is essential to prioritize both innovation and localization in MHE. This requires grounding MHE within the realities of China’s educational context and aligning it with the unique mental health development characteristics of Chinese students. While drawing on successful global practices, it is imperative to move beyond traditional models by exploring innovative formats and methodologies. This includes expanding MHE beyond the classroom into the broader school environment, integrating school-based initiatives with community engagement, bridging offline and online platforms, and shifting from teacher-led instruction toward a participatory model that actively involves educators and fosters student-led experiential learning. The objective is to develop a holistic, multidimensional, and synergistic MHE framework that seamlessly integrates primary, secondary, and higher education characterized by distinct Chinese attributes and designed to meet the psychological developmental needs of contemporary Chinese students. This framework must be both scientifically rigorous and practically effective, embedding mental health into educational practices to provide compassionate support and promote students’ mental well-being.

Moreover, the development of localized assessment and evaluation tools, alongside the continuous refinement of personalized mental health support mechanisms, must align with contemporary trends in MHE. Achieving this goal requires a rigorous commitment to scientific standards and methodological precision while balancing the diverse needs of government, society, parents, and students. It also calls for the strategic integration of emerging technologies and innovative methodologies to create more effective and responsive tools for the early detection of mental health issues. In this context, just-in-time adaptive interventions (JITAIs) offer a promising solution (20). By leveraging daily monitoring technologies, such as biosensors and small-data machine learning algorithms, JITAIs can detect early signs of psychological distress with high precision. This real-time, data-driven approach facilitates timely identification of potential mental health concerns, supports the development of personalized intervention strategies, and ensures that appropriate support is delivered promptly and effectively to meet the unique needs of individual students (21).

### 5.4 Ensuring the quality of mental health professionals in schools

The team of mental health professionals in schools serves as the primary force in delivering and implementing MHE across different educational stages and plays a crucial role in the systematic and integrated advancement of such education. Establishing a highly competent team of mental health professionals is essential for safeguarding students’ mental well-being and ensuring the scientific and effective implementation of MHE at every stage. To address the current challenges related to the allocation of mental health professionals, including insufficient numbers, variable quality, and a lack of coherence across educational stages, targeted efforts need to focus on four key areas.

First, in terms of quantity, the allocation of mental health professionals in schools must strictly comply with national policy requirements to ensure that each educational stage is adequately staffed with qualified personnel. According to the 2023 Special Action Plan, universities should assign full-time mental health professionals at a ratio of no less than one per 4,000 students, with each institution maintaining at least two such professionals. Similarly, each primary and secondary school should appoint at least one full-time or part-time mental health professional, preferably with a background in psychology. Most importantly, dedicated professionals should be assigned to these roles on a full-time basis to guarantee consistent and effective support.

Second, in terms of quality, it is essential to uphold the principle of holistic education by not only maintaining an adequate number of full-time mental health professionals but also ensuring continuous training and specialized career development for all personnel involved in mental health work. In particular, full-time mental health professionals should receive regular supervision and professional guidance to enhance their expertise, support ongoing skill development, and promote advancement toward greater specialization and professionalization. This approach will equip mental health professionals in schools with the specialized knowledge and skills needed to accurately identify students experiencing mental disorders, analyze underlying causes, and design targeted interventions to help them effectively address these issues.

Third, fostering interaction, communication, and collaborative dialogue among mental health professionals in schools across different educational stages is essential. Schools should systematically organize conferences, workshops, and site visits to facilitate the exchange of best practices in MHE. These initiatives not only promote the seamless transfer of student mental health portfolios during transitions between schools but also enable mental health professionals to gain a comprehensive understanding of students’ mental well-being. This, in turn, supports both preventive and intervention-focused approaches to student mental health care. Mental health professionals in schools should collaborate with “administrators and other school staff to establish district policies on the storage and disposal of student mental health portfolios that comply with legal standards and professional ethics” (22). Moreover, enhancing coordination with relevant departments—such as school administrative offices, mental health clinics, and community organizations—will not only broaden educators’ perspectives but also foster a holistic, integrated approach to MHE.

According to the 2023 Special Action Plan, concerted efforts should be made to support the academic advancement and theoretical research capabilities of mental health professionals across all educational stages. The government should facilitate opportunities for these professionals across different educational stages to pursue advanced degrees, such as master’s programs in psychology, social work, and related fields. In 2025, the Chinese government launched a targeted enrollment program encouraging university counselors to pursue doctoral degrees in psychology (23). This initiative aims to cultivate a professionally trained cohort of university counselors specializing in MHE as their primary research focus. The program seeks to provide both theoretical frameworks and practical intervention strategies by aligning its research findings with the psychological challenges faced by Chinese students, thereby addressing these issues more effectively. With advanced academic training, PhD-qualified university counselors will gain a deeper understanding of the underlying causes of students’ mental health issues and will be better equipped to design and implement effective intervention strategies. Consequently, this will enable a greater number of students to access high-quality MHE and promote their mental health more effectively.

## 6 Conclusion

Student mental health has emerged as a globally recognized issue, garnering increasing attention across educational and policy domains. To address this pressing challenge, it is imperative to undertake comprehensive and systematic strategic planning, supported by top-level design, for the integration of MHE and research across primary, secondary, and higher education. Such planning must encompass the development of coherent conceptual frameworks, robust institutional mechanisms, systematic implementation strategies, and the continuous development of mental health professionals. By doing so, educational systems can better respond to the diverse psychological needs of students and foster their holistic development. This paper aims to contribute to global MHE initiatives by outlining integrated approaches across educational stages, embedding MHE within a holistic student development framework, and offering practical guidance for researchers and practitioners seeking to implement scalable mental health systems worldwide.

## Data Availability

The original contributions presented in the study are included in the article/supplementary material, further inquiries can be directed to the corresponding author.
